# A Framework for the Design of Risk-Adjustment Models in Health care Provider Payment Systems

**DOI:** 10.1177/10775587241273355

**Published:** 2024-09-03

**Authors:** Andreea Panturu, Richard van Kleef, Frank Eijkenaar, Daniëlle Cattel

**Affiliations:** 1 Erasmus University Rotterdam, The Netherlands

**Keywords:** risk adjustment, provider payments, alternative payment models, financial incentives

## Abstract

Prospective payments for health care providers require adequate risk adjustment (RA) to address systematic variation in patients’ health care needs. However, the design of RA for provider payment involves many choices and difficult trade-offs between incentives for risk selection, incentives for cost control, and feasibility. Despite a growing literature, a comprehensive framework of these choices and trade-offs is lacking. This article aims to develop such a framework. Using literature review and expert consultation, we identify key design choices for RA in the context of provider payment and subsequently categorize these choices along two dimensions: (a) the choice of risk adjusters and (b) the choice of payment weights. For each design choice, we provide an overview of options, trade-offs, and key references. By making design choices and associated trade-offs explicit, our framework facilitates customizing RA design to provider payment systems, given the objectives and other characteristics of the context of interest.

## Introduction

### Background

Over the past decades, health care regulators and policymakers have made efforts to improve efficiency of health care delivery through reform of provider payment systems. Specifically, efficiency—defined as high-quality care at the lowest possible cost—has been pursued through the introduction of prospective elements into payment models. Although attempts to stimulate efficiency through prospective payment features is far from new, the increasingly pressing challenges facing health care systems have ignited a renewed interest in payment reform. Part of this development is the implementation of various Alternative Payment Models (APMs), including comprehensive population-based payment initiatives such as the Alternative Quality Contract in the United States (Chernew et al., 2011) and Gesundes Kinzigtal in Germany (Hildebrandt et al., 2010), but also bundled payment initiatives targeted at specific medical conditions such as the Bundled Payments for Care Improvement initiative in the United States (Struijs et al., 2020). In addition to fully prospective payments, APMs can also be built (and in practice typically are built) on existing fee-for-service (FFS) architectures with or without risk sharing, as seen in initiatives such as the Medicare Shared Savings Program (MSSP) (McWilliams et al., 2018). By introducing a prospective element, APMs aim to reach their goal of incentivizing efficiency by shifting (some) financial responsibility from payers (e.g., governments or insurers) to health care providers.

A typical feature of prospective provider payments is that they rely on some “normative spending level” for the delivery of a predefined set of health care services to a certain patient population. The normative spending level refers to the level of spending that “ought to be” depending on a providers’ patient population rather than the observed spending level. A key element in the determination of normative spending levels is the correction for systematic differences in the health care needs of providers’ patient populations. This correction, which is commonly referred to as risk adjustment (RA), is crucial for realizing a level playing field for providers and for preventing incentives for undesired provider behavior such as risk selection (McWilliams et al., 2023). Depending on the specific design, however, RA can also introduce unwanted incentives, for instance by linking health care utilization with future payment and thereby reducing cost-control incentives (Ellis et al., 2018), or by perpetuating current inefficiencies and inequities in the delivery of care (Ash & Ellis, 2012; Bergquist et al., 2018). As such incentives undermine the efficiency goal, the design of RA in prospective provider payment models requires careful consideration.

In practice, APMs that shift financial responsibility to providers tend to use “off the shelf” RA models that were originally developed for other purposes, such as predicting spending on a large insured patient population for insurer payments (American Medical Association [AMA], 2019; Cattel et al., 2020). However, the use of RA models in provider payment is not a case of “one size fits all.” The reasons for this are that the source and degree of the risk to be adjusted (i.e., what requires compensation versus what falls under the responsibility of providers) as well as the scope of actions that are to be encouraged or discouraged (e.g., in terms of risk selection and cost control) can differ across settings. It is therefore imperative to customize RA models to the specific characteristics of the context in which the payment model is (to be) applied.

The design of RA for provider payment comes with many choices and difficult trade-offs between incentives for risk selection and cost control. Despite a growing literature, no comprehensive framework exists that synthesizes design choices and associated trade-offs, and that can serve as a toolkit for customizing RA to provider payments and the objectives and other characteristics of the context of interest. By exploring RA specifically in the context of provider payment models with prospective elements, this article aims to develop such a framework. After describing the contribution of this article to the literature and defining key concepts (section “Definitions of Key Concepts”), section “Three Criteria for the Design of RA Models” introduces three criteria for the design of RA models. Next, section “A Framework for the Design of RA Models” presents the framework of design choices, followed in section “Discussion” by a discussion of various normative and practical aspects related to these choices.

### New Contribution

The existing literature already contains important conceptual contributions with respect to RA design. Specifically, Ellis et al. (2018) delve into the design of RA models for insurer payments, and Iezzoni (2012) provides a handbook on RA for measuring health care outcomes, also offering some insights into the critical role of RA in the design of provider payments. Despite these contributions and an expanding literature on this topic, currently no integral framework exists for tailoring RA design to provider payment and essential features of the context. By synthesizing, extending upon, and applying insights from existing literature, this article develops such a framework.

First, we conducted a literature review with snowballing, combined with expert consultation. The literature review covers prior work on the design of RA models in both provider and insurer contexts as well as (empirical) literature on selection and cost-control incentives (such as upcoding) potentially introduced by RA, that may undermine the goals of the payment system. Considering the broad scope and diverse nature of the study topic, which would be challenging to adequately capture with specific search terms, we deliberately opted for an exploratory and flexible methodology over a systematic literature review. To address the concern that potentially relevant literature might be missed using this approach, we followed an iterative process involving multiple meetings with experts in the field of RA and payment systems, including the international Risk Adjustment Network, at different stages of the framework development. During these meetings, the framework was presented to and discussed in detail with the experts, focusing on clarity and potential gaps in the framework. No new design elements, options, or trade-offs emerged from these discussions, providing us confidence that saturation had been reached.

Second, the collected information from the literature review and expert consultations was synthesized to develop the framework. From this synthesis, three criteria for the design of RA models emerged. We then clustered the identified design choices and associated options and trade-offs in two overarching dimensions: (a) the choice of risk adjusters and (b) the choice of payment weights. By making design choices and associated trade-offs explicit and discussing the essential role of context, with our framework we offer a practical toolkit for the design and implementation of RA models for prospective provider payment models.

## Definitions of Key Concepts

### Prospective Payment, Financial Responsibility, and Normative Spending Levels

The potential effects of (changes in) provider payment models have been well described (McGuire, 2000, 2011). One conclusion that can be drawn from the existing literature is that traditional FFS payment models may undermine efficiency. By traditional FFS payment models we mean models that reimburse providers for each discrete service provided, placing most of the financial responsibility for health care spending at the payer (e.g., a government or an insurer). As a result, when fees exceed the marginal cost of providing additional services, FFS results in perverse incentives that might stimulate providers to prioritize volume over value (i.e., efficiency).

Unlike traditional FFS models, APMs shift financial responsibility from payers to providers. How and to what extent this occurs depends on the type of APM and its specific design. Under the right circumstances, shifting (some) financial responsibility can increase incentives for cost control and result in actions such as increased prevention activities and coordination of care, consequently contributing to the efficiency objective of APMs (Cattel et al., 2020; Porter & Kaplan, 2016). Financial responsibility can be shifted at the level of specific medical conditions (i.e., bundled payments) or at the population level (i.e., population-based payments). In addition, it can involve a full shift of responsibility (e.g., in fully prospective models) or a partial shift (e.g., shared savings/losses models built on existing FFS architectures).

Regardless of the type of model, any shift of financial responsibility to providers requires that the payer determines the normative level of spending, reflecting the appropriate spending level that “ought to be” given the health care needs of a population and the goals of the APMs. At the individual level, the normative spending level is defined as “the appropriate spending level for individual *i* for period *t* and the set of health care services *s*, before the start of *t*.” At the population level, it simply equals the sum of the individual-level normative levels in population p. It is important to note that the normative spending level does not necessarily refer to the absolute or optimal spending level, but rather to the spending level considered appropriate given the efficiency level/objectives pursued by the APM. Moreover, this normative (or appropriate) spending level typically differs from the observed spending level, as the latter may not accurately reflect the true health care needs of patients and capture potential inefficiencies from the observed health care system, which the APM aims to address.

### Sources of Spending Variation and the Role of RA and Risk Sharing

When establishing normative spending levels, it is important to consider three sources of spending variation: (a) systematic variation driven by *factors that are beyond the control of providers* for which providers *should not* be held responsible (“compensation factors,” henceforth called “C variables”), (b) systematic variation driven by *factors that providers can influence* for which providers *should* be held responsible (“responsibility factors,” henceforth called “R variables”), and (c) random variation.

When shifting financial responsibility to providers, it is important to prevent providers from facing (excessive) risks that they cannot influence as this can undermine the efficiency goal of the APM. To prevent turning providers into insurers, providers need to be protected from financial risk due to random variation in health care spending (Anderson & Weller, 1999; van Barneveld et al., 2001).

In practice, APMs therefore often apply some form of risk sharing such as stop-loss reinsurance schemes covering most or all spending of the most expensive patients, or limiting providers’ financial responsibility to a certain share of savings (when actual spending is less than the prospective payment/spending target) or losses (when spending exceeds the prospective payment/target) (van Barneveld et al., 2001). However, because our focus is on the *predictable* share of spending variation, this article considers the form and extent of risk sharing as given.

Of the predictable part of spending variation, only variation due to R variables should fall under the responsibility of providers; variation due to C variables should be compensated for in order to protect providers from predictable financial consequences outside of their control. To do so, APMs often use some form of prospective RA. As part of this, decisions on which variables are considered C and which R are required. It is crucial to consider both demand-side utilization factors related to patient characteristics such as predisposition (e.g., attitudes, beliefs), ability (e.g., income, health insurance), and illness level, as introduced by Andersen and Newman (2005), as well as supply-side utilization factors related to the performance of healthcare providers. Typical C variables are illness level and other factors that relate to a patient’s health needs. Typical R variables are supply-side factors related to the providers’ performance, such as suboptimal provision of care.

Some utilization factors, however, are more difficult to categorize as C or R variables. These are factors that are based on interactions between patients, providers, and payers, that can result in health care utilization that does not match a patient’s health needs (Arrow, 2004; McGuire, 2000; Vermaas, 2006). In addition, as a result of these interactions, these factors are not always fully under the control of providers. For example, it is not always clear whether overconsumption due to patient preferences or lifestyle should be classified as R or C. The answer to this question depends on the context. Classifying this factor as R may disadvantage providers who face difficulties in influencing their patients or those who place a high value on their patient’s preferences. However, classifying this factor as C may undermine the efficiency goal of APMs. As a result, it is often unclear whether (spending variation resulting from) such factors should or should not be the financial responsibility of providers. Other factors that may be challenging to label in this regard are discretionary supply-side variables that may increase both costs (at least in the short term) *and* quality, such as “investing in high-quality diabetes care.” In such cases, whether these variables are labeled as C or R will depend on the scope and goals of the APM.

The nature of and degree to which spending variation resulting from C variables is to be compensated for forms the starting point of an RA model. The degree of spending variation resulting from C variables is context-dependent, and can be determined by the care package, the patient population to which the payment model applies and the form and the extent of risk sharing. Ceteris paribus, a comprehensive care package and diverse patient population relate to higher spending variation, while increased risk sharing relates to lower spending variation. Whether this spending variation is predictable or random, and consequently the extent to which RA can be used, also depends on these factors. For example, care for long-term manageable diseases such as diabetes and chronic obstructive pulmonary disease (COPD) is found to associate to a relatively large degree of predictable spending differences related to patient health needs (typical C variable) (Chang et al., 2010). In contrast, acute care is less predictable and typically associates more strongly with random variation, which is beyond the reach of RA. Moreover, highly heterogeneous patient populations may be more unpredictable and harder to risk-adjust (Porter & Kaplan, 2016). Finally, we recognize that risk sharing is a generic measure that is difficult to target specifically toward unpredictable variation, and that therefore will likely also compensate—to some extent—for systematic variation. This means that the need for RA to compensate for predictable variation will also depend on the level and form of risk sharing (here assumed as given). If providers are already compensated through risk sharing for C spending variation, RA will no longer be necessary. Also, if RA fails to compensate for some systematic variation, risk sharing could be used to make up for it. To conclude, although the aforementioned aspects are important for RA design in practice, due to their context-dependent nature, our conceptual framework considers them as given and does not delve into their specific design.

## Three Criteria for the Design of RA Models

The general goal of RA in APMs is to compensate health care providers for spending variation due to C variables, while keeping them responsible for spending variation due to R variables. This goal implies two key criteria for the design of RA models: (a) adequate compensation for C variables and (b) no compensation for R variables. A third important criterion, resulting from our synthesis, is feasibility. Table 1 provides a summary on the three key criteria for the design of RA models, supported by key references in the third column. These criteria are further discussed below.

**Table 1:** The Criteria for the Design of RA Models.

Criteria	Operationalization	References^a^
1. Adequate compensation for spending variation due to C variables	1. Should compensate for C variables that are relevant in the light of potential risk selection actions by providers	Ellis et al. (2018), Ellis (1998), Iezzoni (2012), Isaksson et al. (2018), Newhouse (1994), van de Ven and van Vliet (1992), van de Ven and Ellis (2000), Werbeck et al. (2021)
	2. Should compensate for C variables that vary across provider populations to prevent selective participation	Einav et al. (2022), Levy et al. (2018), Liao et al. (2020)
2. No compensation for spending variation due to R variables	1. Should not compensate for existing ‘status quo’ inefficiencies	Ash and Ellis (2012), Bergquist et al. (2018)
	2. Should not compensate for new volume and/or price-based inefficiencies	Ellis et al. (2018), Geruso and Layton (2020), Geruso and McGuire (2016), Iezzoni (2012), Newhouse (1994), van de Ven and Ellis (2000)
3. Feasibility	1. Should have data availability	Ellis et al. (2018), Iezzoni (2012), van de Ven and Ellis (2000)
	2. Should be accepted among all stakeholders^b^	Ellis et al. (2018), Iezzoni (2012), van de Ven and Ellis (2000)

a Note that this reference list is not exhaustive; the aim is to provide support for each criterion and its operationalization. ^b^Ensuring stakeholder acceptability is vital for the effective implementation of the RA model, particularly among providers, to ensure their participation in the program.

### Criterion 1: Adequate Compensation for C Variables

To avoid selection problems, RA should adequately compensate for C variables. In this study, based on prior literature, we distinguish between two selection problems that can occur when this criterion is not met. Selection Problem 1 is referred to as “risk selection” and concerns actions to attract (deter) healthy (unhealthy) patients with relatively low (high) expected spending (Ellis, 1998; Newhouse, 1994; Ellis et al., 2018; Iezzoni, 2012; van de Ven & Ellis, 2000; van de Ven & van Vliet, 1992). This type of selection implies that providers assess whether the payment will be sufficient (i.e., neither too low nor too high) to appropriately help a given patient with a given health problem. It is reasonable to assume that providers have significant possibilities to engage in risk selection as they have direct contact with patients and detailed knowledge of their patients’ health (Martinussen & Hagen, 2009). For example, orthopedic surgeons under the Comprehensive Care for Joint Replacement (CJR) bundled payment program in the United States were found to exclude high-risk patients from operations (identified by body mass index, diabetes control, or other predictors of complications) for which payment was estimated to be insufficient to cover the costs of care services included in the bundle (Humbyrd et al., 2019). Another example would be providers selecting a practice location in an affluent suburban area, in an attempt to attract *healthy* patients with below-average expected spending (Isaksson et al., 2018). Risk selection is undesired because it has negative effects on the accessibility and quality of care for patients who are predicted to be unprofitable, and may also lead to (increased) disparities in the accessibility and quality of care between profitable and unprofitable patient groups (e.g., Werbeck et al., 2021).

Selection Problem 2 concerns “selective participation” in the APM by providers based on their expectations about the (un)profitability of their patient population under the APM. This is particularly relevant when provider participation is voluntary, as is often the case in practice. If there is insufficient compensation for C spending variation between providers, providers with a relatively high share of patients with high expected spending due to C variables will be less likely to participate and more likely to opt out (Einav et al., 2022). Selection on (expected) gains, also referred to as Roy selection by Heckman and Honore (1990), can be undesirable for two reasons: (a) the APM might not reach all the providers it intends to reach (and thereby not realize its full potential in terms of increasing efficiency), and (b) participants with relatively healthy patient populations might gain from participating without changing behavior, undermining the required behavioral change for increasing efficiency.

The extent to which a lack of compensation for C variables within RA models is problematic depends on the extent to which there is room for the aforementioned selection problems. For Selection Problem 1, this depends on the possible selection actions by providers in a given context. More specifically, a lack of compensation for C variables across patient groups is especially problematic if providers have possibilities to selectively attract and deter certain patient groups (e.g., Ellis, 1998).This is most challenging when population is based on patient choice, as opposed to being geographically determined. For Selection Problem 2, C spending variation across providers can result in participation imbalances when the risk profile of populations differs (or is perceived to differ) among providers. When providers cannot select into/out of the program, this selection problem is less of a concern (Levy et al., 2018; Liao et al., 2020). Yet, even if participation in the sense of partaking yes/no is no longer a concern, getting each provider to participate actively and invest in better care might still be challenging if providers are, or have the feeling of, being under/overcompensated for C variables. Finally, we emphasize that while (perverse) financial incentives and the possibility of selective/inefficient behavior may be present, providers have an intrinsic motivation to avoid causing harm. Thus, whether and how providers act on financial incentives depends on how they weigh these factors against their intrinsic motivation.

### Criterion 2: No Compensation for R Variables

To prevent inefficiencies, RA should not compensate for R variables. When providers are compensated for R spending variation, this can give rise to perverse financial incentives that can materialize into inefficient behavior and effects. Our synthesis revealed a distinction between two efficiency problems that can occur when providers are compensated for R variables: (a) the perpetuation of *existing* inefficiencies and (b) the creation of incentives for *new* inefficiencies.

The first efficiency problem can be defined as “status quo bias” and refers to the difference between patients’ observed health care utilization and the utilization required to meet their actual health needs (Ash & Ellis, 2012). Several studies have provided evidence for such bias in observed health care utilization and have shown the importance of explicitly addressing it when designing RA models (Ash & Ellis, 2012; Bergquist et al., 2018). To understand this bias, consider a provider who has performed unnecessary treatments in the past. When RA is based on historical utilization and spending, as is typically the case in practice, such inefficient provision patterns risk being perpetuated as the provider will be compensated for it. This undermines the efficiency goal of the APM and may also result in the second efficiency problem discussed below. In addition, it might be considered unfair when providers are financially “rewarded” for inefficient behavior in the past.

The second efficiency problem refers to creating incentives for *new* inefficiencies. RA can create a direct link between diagnoses, utilization, or spending and future payments, which subsequently could lead to a reduction in incentives for volume- and price-control (Ellis et al., 2018; Geruso & McGuire, 2016; Iezzoni, 2012; van de Ven & Ellis, 2000). Finally, reduced incentives for volume control can result in behavior such as overprovision, and reduced incentives for price control can materialize in so-called upcoding, that is, an actual shift to higher-priced treatments/services resulting in higher spending *or* fraudulent behavior, where services with higher payment are falsely claimed to have been provided to patients (Dafny, 2005; Anthun, 2021; Bäuml, 2021; Geruso & Layton, 2020). Incentives for both types of undesired behavior can arise when using *endogenous* risk adjusters based on (prior) diagnoses, utilization, or spending, that is, risk adjusters that can be influenced by providers. Having said that, the extent to which perverse incentives are problematic depends on the possible actions in terms of changing practice patterns and upcoding, and the probability and potential effects of these actions in a given context.

### Criterion 3: Feasibility

A crucial third criterion that emerges from our synthesis is feasibility. For the purpose of our framework, we discern between feasibility with respect to (a) data availability and (b) stakeholder acceptability, acknowledging that other feasibility challenges (such as the time lag between data collection and its use for payment purposes) may exist. First, the availability of data and an adequate data infrastructure for the purpose of RA can be very challenging to obtain and realize in practice (Ellis et al., 2018; Iezzoni, 2012; van de Ven & Ellis, 2000). For instance, individual-level data are often not available, limiting many countries to using data on an aggregated level (Ellis et al., 2018). Second, the endorsement and acceptability of the RA design across all stakeholders (e.g., patients, providers, payers, and regulators) can also be a complex task (Epstein & Cumella, 1988; van de Ven and van Vliet, 1992). Acceptability is likely to be influenced by several factors that can vary across settings. Examples of such factors are the privacy-sensitivity of the information being used and the executability/explainability of an RA model. Furthermore, stability can also affect acceptability: that is, a RA model that ceteris paribus (re)produces more or less the same payment weights over time is more likely to be accepted by stakeholders.

## A Framework for the Design of RA Models

This section develops a conceptual framework for the design of RA for prospective payment models. We distinguish between design questions, associated options, and key considerations and trade-offs with respect to (a) the choice of the risk adjusters and (b) the choice of the payment weights attached to these risk adjusters. These two main themes are considered as distinct steps in the design of RA models due to their unique key considerations and trade-offs and are therefore discussed separately in section “The Choice of Risk Adjusters” and “The Choice of Payment Weights,” respectively (their relationship is further explored in section “The Interconnection Between Design Choices for Risk Adjusters and Payment Weights”). The aim of the framework is to make the design choices explicit and indicate how the aforementioned criteria play a role.

### The Choice of Risk Adjusters

Table 2 summarizes the questions, options, and considerations regarding the choice of risk adjusters. The first column contains three main design questions: (a) What type of information are risk adjusters based on; (b) Which time/base period does the information pertain to; and (c) How to design risk adjusters? The second column lists the options for each design choice, while the third column outlines the various trade-offs. On one hand, we evaluate trade-offs with respect to incentive effects related to C and R variables: (a) selection incentives, (b) perpetuation of prior inefficiencies, (c) incentives for volume control, and (d) incentives for price control. Here, we do not distinguish between the two selection problems, that is, risk selection across patients and selective participation among providers, as RA affects them in the same way. On the other hand, we also consider trade-offs with respect to so-called “technical complexity,” which is determined by elements such as the number of parameters in the model, potential data alterations, and the estimation method. In this framework, we distinguish technical complexity from subjective feasibility elements such as acceptability, which, given their context-dependent nature, are not considered when determining conceptual trade-offs. Finally, the fourth column contains references to a set of selected papers that discuss the specific design questions, options, and trade-offs in detail.

**Table 2:** Key Design Aspects Regarding the Choice of Risk Adjusters.

Design question	Options	Direction of incentive effects	References^e^
**1. What type of information are risk adjusters based on?**	Demographic information: age, gender	• Reduce selection incentives^b^	Ellis et al. (2018), Iezzoni (2012)
	Socioeconomic status (SES) information: e.g., poverty, work status, disability, education, ethnicity, residence		Ash et al. (2017), Durfey et al. (2018), Ellis et al. (2018), Iezzoni (2012), Jha (2014)
	Subjective (health) information: e.g., self-perceived health, patient preferences concerning quality of life, expectations on the healthcare system, demand elasticities^a^		Ellis et al. (2018), Iezzoni (2012), Lamers (1998), Withagen-Koster et al. (2020)
	Diagnostic information: e.g., diagnosis-based cost groups (e.g., DCGs or HCCs)	• Reduce selection incentives^b^ • Can perpetuate prior inefficiencies • Reduce incentives for volume control	Anthun (2021), Bäuml (2021); Dafny (2005), Ellis et al. (2018), Iezzoni (2012), Lamers (1998), Pope et al. (2004), Pope et al. (2011)
	Utilization information: e.g., pharmacy-based cost groups (e.g., PCGs), the use of specific care such as durable medical equipment, hospitalization		Ellis et al. (2018), Gilmer et al. (2001), Iezzoni (2012), Lamers (1999), Lamers and Van Vliet (2003)
	Clinical information: e.g., severity of illness (e.g., tumor grade), comorbidities (e.g., obesity), the complexity of the procedure, end of life probabilities		Ellis et al. (2018), Hughes et al. (2004), Iezzoni (2012), Kapur et al. (2003)
	Spending information (lagged): e.g., total past spending, spending by type of service	• Reduce selection incentives^b^ • Can perpetuate prior inefficiencies • Reduce incentives for volume control • Reduce incentives for price control	Ellis et al. (2018), Iezzoni (2012), van Kleef and van Vliet René (2012)
	Supply-side information: e.g., number of providers, prices of services	• Reduce selection incentives^b^ • Can perpetuate prior inefficiencies • Reduce incentives for price control	Constantinou et al. (2022), Gravelle et al. (2003)
**2. What time period (base period) does the information pertain to?**	Prospective: the period prior to prediction	Incentive effects unknown (and probably context dependent)^c^	Adams Dudley et al. (2003), Ellis et al. (2018), García-Goñi et al. (2009), Iezzoni (2012)
	Concurrent: the period that coincides with the prediction period		
**3. How to design the risk adjusters?**	Specification of the scale of measurement: e.g., continuous, logarithmic, binary, quadratic/polynomial	• Reduce selection incentives^b^ • Reduce incentives for volume control • Increase technical complexity^d^	Ellis et al. (2018), Hollenbeak (2005), Iezzoni (2012)
	Operationalization of risk adjusters: e.g., consider *conditions* such as the width of age bands (e.g., include age as 10-, 5-, or 1-year classes), or the type and number of medication prescriptions needed to trigger classification in a risk class, and *hierarchies/restrictions* for people that may trigger multiple risk classes		Douven, Remmerswaal, & Mosca (2015), Ellis et al. (2018), Eijkenaar et al. (2018), Horn et al. (1984), Iezzoni (2012), Lemke et al. (2020), Pope et al. (2011), Politzer (2024)
	Interactions between risk adjusters: e.g., between age and gender, or diagnoses		Buchner et al. (2017), Ellis et al. (2018), Iezzoni (2012), Pope et al. (2011), Van Veen et al. (2018)

a Risk adjusters that are based on subjective (health) information are considered to be exogenous just like demographic or SES information, as they cannot be influenced by health care providers. However, note that exogenous does not necessarily mean valid. Validity is questionable in the case of subjective health information case. ^b^ A reduction of “selection incentives” refers to a reduction of incentives for providers to (a) under/overserve specific risk types (i.e., risk selection) and (b) select in and out of the APM program (i.e., selective participation). ^c^ The impact of a risk adjusters’ timeframe on selection and efficiency incentives can vary depending on the context. ^d^ Technical complexity is determined by factors such as the number of parameters in the model, potential data alterations, and potential estimation methods. Technical complexity relates to the conceptual elements of feasibility that are consistent across settings. ^e^ Note that this reference list is not exhaustive, as the aim is to provide support for each for each design question and associated option and incentive effects.

#### What Type of Information Are Risk Adjusters Based on?

The first design question entails the type of information to be used as a basis for risk adjusters. To maximize the degree to which C spending variation is captured by the RA model, it is common to use risk adjusters based on different types of information (Ellis et al., 2018; Iezzoni, 2012; van de Ven & Ellis, 2000). For example, demographic and socioeconomic information is exogenous (i.e., not prone to providers’ influence) and therefore do not maintain or introduce volume- or price-related perverse incentives (Ash et al., 2017; Durfey et al., 2018). However, adjusters based on that type of information are unlikely to sufficiently mitigate selection problems as their predictive power is generally relatively low (Brown et al., 2014). In contrast, information on (prior) diagnoses, utilization, and spending make up endogenous adjusters that, while known to be highly predictive of C-type spending (Pope et al., 2004), can capture and perpetuate inefficient practice (i.e., status quo bias) and introduce incentives for new volume- or price-based inefficiencies by creating a link between utilization/diagnoses and normative spending/payment (Ellis et al., 2018). More specifically, diagnostic- and utilization-based adjusters can lead to reduced incentives for volume control while spending-based adjusters can *also* weaken incentives for price control. Since diagnostic- and utilization-based adjusters (and the compensation that is based on them) are directly linked to the provision of care, under these adjusters there is a stronger incentive for volume inefficiencies. Spending-based adjusters, however, are directly linked to (historical) health care costs and can therefore lower both price and volume control incentives. So, the use of endogenous information for developing risk adjusters comes with a trade-off between selection and efficiency, with the optimal trade-off depending on contextual factors such as the possibilities for risk selection and for volume- and price-control.

#### What Time Period (Base Period) Does the Information Pertain to?

The second design question regards the time or “base” period that the information used for the risk adjusters pertains to. In this respect, a distinction can be made between concurrent and prospective adjusters, which in practice are often combined in a single model (Ellis et al., 2018; Iezzoni, 2012; van de Ven & Ellis, 2000). Remarkably, there is hardly any empirical literature on the relative performance of these options in terms of incentives for selection and efficiency. It can be argued that prospective adjusters come with higher data requirements than concurrent adjusters, but the incentive effects relative to concurrent adjusters are a priori unclear. While concurrent adjusters may ceteris paribus result in a better overall fit (e.g., in terms of a higher R-squared from a regression model), this better fit does not necessarily mean that concurrent adjusters result in lower predictable profits or losses than prospective adjusters. Concurrent adjusters may have a better fit simply because they capture more random (i.e., unpredictable) spending variation as they are based on information pertaining to the same period as the spending data on which the model is estimated. It is therefore not necessarily the case that concurrent adjusters lower selection incentives more than prospective adjusters. A similar argument holds for incentives for volume- and price-control: while payments linked with concurrent adjusters can create incentives for fraudulent/inefficient coding, the extent to which prospective adjusters result in fewer or no such incentives remains unclear (Adams Dudley et al., 2003). Theoretically, the choice of time lag between the base and current period for risk adjusters based on diagnostic-, utilization- and spending-information may play a role in the trade-off between predictiveness and negative incentives with respect to volume- and price-control. As such, the negative incentives could be reduced by increasing the length of base period. Nevertheless, empirical research is required to confirm this.

#### How to Design the Risk Adjusters?

The third design question relates to the specification and operationalization of the risk adjusters that are to be used in the RA model. First, the risk adjusters need to be specified in terms of their assumed relationship with health care spending. In practice, risk adjusters are typically included as a (collection of) binary variables with associated “risk classes” in which patients are classified (Ellis et al., 2018). Second, the risk adjusters/classes should be operationalized considering the use of certain conditions, hierarchies, and restrictions for classification of patients, as well as possible interactions between risk adjusters (Ellis et al., 2018; Iezzoni, 2012). For example, classification of patients in certain risk classes can be conditioned by yes/no surpassing predefined thresholds, an approach that is often used for medication-based risk adjusters in the form of a certain type and number of prescriptions needed to trigger classification in a risk class (Politzer, 2024). Although previous work has highlighted volume-related perverse incentives associated with using thresholds (Douven, Remmerswaal, & Mosca, 2015), Politzer (2024) discovers that the use of *explicit* and *carefully defined* thresholds for medication-based adjusters can both improve the model fit, thereby reducing selection incentives, *and* decreasing volume-related perverse incentives related to the respective adjuster. However, this comes at a cost of increased technical complexity. Next, classification in risk classes can also depend on the use of hierarchies and restrictions (Ellis et al., 2018). Hierarchies are typically derived from classification systems like the International Classification of Disease (ICD) and are often applied to diagnosis-based risk adjusters in such way that risk classes capture only the most severe and costly condition categories. Restrictions can be considered for subgroups of people that might qualify for classification in multiple risk classes. While these subgroups could be restricted to just one risk class to reduce technical complexity, allowing certain subgroups to be classified in multiple risk classes might improve the predictive performance of the RA model and as such, reduce selection incentives. This may be especially the case for people with comorbid conditions (Eijkenaar et al., 2018). Finally, interactions between risk classes can be used to capture nonlinear patterns (Buchner et al., 2017; Ellis et al., 2018, Van Veen et al., 2018). This can be particularly useful is case of comorbid conditions. For example, having both diabetes and heart failure may result in higher spending than would be estimated when using separate risk adjusters for both diseases (Pope et al., 2011). Although including an interaction term between such disease pairs can improve fit and reduce selection incentives, it also increases the technical complexity of the RA model.

### The Choice of Payment Weights

Table 3 summarizes the questions, options, and considerations regarding the choice of payment weights. Ideally, the payment weights in an RA model *fully* and *exclusively* capture spending variation due to C variables. In finding appropriate payment weights, decision-makers are confronted with three main design choices: (1) the selection of an estimation sample that is as representative as possible for the setting in which the RA model is applied, (2) the selection of data interventions on the estimation sample to improve its representativeness, and (3) the selection of a method for deriving the payment weights, which involves deciding which risk adjustors to include and which optimization criterion to use when estimating these weights. These choices are further discussed below.

**Table 3.** Key Design Choices Regarding the Choice of Payment Weights.

Design question	Options	Key considerations and Trade-offs	Key references^a^
**Which estimation sample?**			
1.a. Which patient population?	Patient populations: e.g., population targeted by payment model, population of other provider(s), national/regional population	• Estimation sample should match the population of interest and their normative spending level in year *t* as closely as possible	Douven, McGuire, & McWilliams (2015), Ellis et al. (2018), Iezzoni (2012), Rose et al. (2016)
1.b. Which prediction time period?	Time period: e.g., spending data from year *t*, historical spending data (e.g., how many prior years to include, how to weight each year)		
**Which data interventions?**			
2.a. Which interventions on the patient population?	Representativeness year *t*: e.g., correct for changes in the population composition	• Estimation sample should match the population of interest and their normative spending level in year *t* as closely as possible • Modifications of the spending data require additional information and thereby increase technical complexity	Ellis et al. (2018), Iezzoni (2012)
	Representativeness population of interest: e.g., exclude partial-year patients, outliers		
2.b. Which interventions on the spending data?	Representativeness year *t*: e.g., determine the risk-bearing spending and correct for cost inflation, regional trends, changes in treatment technologies		Ash and Ellis (2012), Bergquist et al. (2018), Ellis et al. (2018), Iezzoni (2012)
	Representativeness normative spending levels: e.g., correct for status quo bias (R variation)		
**How to derive the payment weights?**			
3.a. Including R variables in the estimation model?	Correction for omitted-variable bias: include R adjusters in RA model to improve the payment weights of C adjusters	• What you are actually optimizing statistically should match what you want to achieve (i.e., true objective function) • More advanced objective functions/estimation methods increase technical complexity	Schokkaert and Van de Voorde (2004); Schokkaert and Van de Voorde (2006)
3.b. Which optimization criterion (e.g., estimation method)?	Standard fixed optimization criterion: linear least squares (ordinary least squares, OLS)		Ellis et al. (2018), Iezzoni (2012)
	Other fixed optimization criterion: e.g., maximum likelihood (e.g., generalized linear models, GLM), moment conditions (e.g., generalized methods of moments, GMM), multiple fixed criteria (e.g., two-part models)		Duan et al. (1983), Ellis et al. (2018), Iezzoni (2012), Iommi et al. (2022), Jones (2010)
	Customized optimization criterion (e.g., constrained regression, machine learning)		Ellis et al. (2018), Rose (2016), van Kleef et al. (2017, 2020)

a Note that this reference list is not exhaustive, as the aim is to provide support for each for each design question and associated options and trade-offs.

#### Which Estimation Sample?

A representative estimation sample is required for accurate payment weights estimation. This includes the selection of a patient population and of an appropriate time period for the corresponding spending data (Ellis et al., 2018; Iezzoni, 2012). To adequately compensate for C spending variation, the estimation sample ideally captures the population of interest to the payment model *and* the normative spending levels for each individual in this population in year *t*. In practice, however, data on the population of interest may not be readily available, which is why similar patient populations from other providers are often considered (Ellis et al., 2018). Alternatively, an estimation sample can be selected from national/regional registries. Similarly, since spending data from year *t* is not available before the start of year *t*, historical data are used in practice (Rose et al., 2016). Questions like “how many prior years to include” and “how to weigh each year” then need to be answered to ensure that the historical spending data are as representative as possible for the spending of the population of interest in year *t*. For example, a recent APM initiative in the Netherlands determined its benchmark spending levels on the basis of the 3-year weighted average prior spending with more recent years weighted more heavily (Hayen et al., 2015). Finally, despite it being theoretically possible to establish normative spending levels using clinical guidelines or expertise, in practice, researchers typically rely on observed spending levels as a proxy for health care needs. Observed spending levels, however, may be biased as they reflect the current flawed health care state that reform seeks to address, as noted by Ash and Ellis (2012), particularly if the setting from which the data is derived operates less efficiently. This can be illustrated with a real-world example from a different context (health plan payment instead of provider payment): Medicare Advantage (MA) plans rely on FFS Medicare risk weights to set their capitation payments (Newhouse et al., 2015). However, as MA operates under a capitated payment model and Traditional Medicare (TM) under FFS, different dynamics of care delivery and provider incentives are at play. In this case, besides ensuring the representativeness of spending data for the target population, it is also essential to account for the efficiency differences between the TM model (reflected by the FFS spending data used for estimating the payment weights) and the MA model, where these weights are intended to be applied.

#### Which Data Interventions?

When the estimation sample is not representative for the population of interest, or of its true health care needs and the efficiency level targeted by the APM in year *t* (i.e., normative spending level), data interventions on the patient population and/or spending data should be considered. To improve the match between the observed population and the population of interest in year *t*, differences in the composition of the observed population can be corrected and certain individuals could be excluded (e.g., outliers, patients only registered for a part of the year, mistakes in data collection, and values capturing random variation or predictable variation with no data for the necessary adjuster) (Ellis et al., 2018; Iezzoni, 2012). Next, several interventions can be considered to improve the match between observed spending and normative spending in year *t*. First, it is important to determine the C spending variation that RA should compensate for. For instance, if the payment model has a risk-sharing component that fully covers the C spending of patients with extremely high spending, the RA model only needs to compensate for the spending up until the point where the risk-sharing begins (van Barneveld et al., 2001). Corrections for inflation, (regional) treatment trends, and/or the availability of new treatment technologies can then be used to make the spending data that requires RA representative for period *t* (van Kleef & Reuser, 2021). Second, direct intervention on the spending data can be considered to improve the match between observed spending and normative spending levels in the relevant setting (Bergquist et al., 2018). We discuss the mechanics of this intervention in more detail below.

As previously noted, the use of observed spending for estimating RA models is likely to introduce status quo bias in the payment weights (Ash & Ellis, 2012). Unlike inefficiencies related to volume-and price-control, which enter the model and might be perpetuated via the inclusion of diagnosis-, utilization-, and spending-based risk adjusters, inefficiencies in health care utilization between population subgroups (also referred to as “inequities”) sneak into the payment weights via the spending data. Assume that patient groups with a low socioeconomic status (SES) underutilize care while high SES groups overutilize care. Regardless of the source of this inequity, it reflects a mismatch with the actual patient needs, that is reflected in the observed spending and that should not be maintained. However, there is no straightforward solution to address this inequity. Including SES as risk adjuster may perpetuate this status quo bias by compensating for the inappropriate provision of care across groups (Chien et al., 2017), while excluding a SES-based adjuster merely makes the underpayment less visible and does not prevent penalizing providers who care for patients with a low SES (Ellis et al., 2018). Such inequities should therefore be addressed via the payment weights. Specifically, along with the inclusion of SES-based adjusters, to ensure that providers are not penalized for serving more patients with a low SES and vice versa (McWilliams et al., 2023), this would involve intervening on spending data to correct for the inequity between SES groups (Bergquist et al., 2018). An example of this is to inflate spending for groups known to underuse care. Although such modifications might improve the match between observed spending and the needs of the target population, they also lead to an increase in technical complexity and data requirements (e.g., to determine the level of under- and overutilization for specific SES-groups).

#### How to Derive the Payment Weights?

The final design steps pertain to deriving the payment weights using the established set of risk adjusters (Table 2) and estimation sample. Before turning to the estimation process, it is important to first conduct a final check for potential bias in the model. One important issue to consider is the (potential) correlation between C variables and R variables, as pointed out by Schokkaert and Van de Voorde (2004, 2006). In the presence of such correlation, excluding R variables from the RA estimation model can introduce so-called omitted-variable bias and compromise the appropriateness of payment weights. For example, the authors label the variable “being chronically ill” as R, due to concern that this can to some extent be manipulated by sickness funds. This variable is, however, also likely to capture important information about a patient’s health needs. Excluding this variable from the model could therefore bias the estimated payment weights for C variables and create large incentives for risk selection, which is in line with their findings (Schokkaert & Van de Voorde, 2006). To address this issue, Schokkaert and Van de Voorde propose a so-called “explicit approach” that consists of the following two steps: (a) estimate the payment weights for C variables with a model that also includes R variables and (b) neutralize the coefficients for R variables when calculating the normative spending level. Nevertheless, even though this approach (i.e., including R variables) can improve the payment weights, it increases technical complexity as it is challenging to differentiate between the C and R components of risk adjusters.

After determining which risk adjusters to include (i.e., whether to include R variables), the final step is to estimate the actual payment weights. In practice, payment weights are typically estimated using fixed statistical objective functions that can be estimated using ordinary least squares (OLS) (Ellis et al., 2018). However, it is unclear whether this approach yields the most appropriate payment weights. First, the distribution of the spending is an important factor in determining the most appropriate estimation method. As spending data tend to be skewed, it may be useful to explore different functional forms. Other statistical methods such as generalized linear models (GLMs) can be used to handle transformations of the spending data, enabling more flexible functional forms (e.g., nonlinear) (Duan et al., 1983; Jones, 2010). Alternatively, in scenarios with many zero-spenders, estimation methods such as two-part models may be more suitable (Jones, 2010). Second, and most importantly, as discussed in this article, APMs serve an economic purpose and therefore, the objective of RA is centered on promoting efficiency. This however, as also argued by Layton et al. (2018) and Glazer and McGuire (2002) often cannot be achieved through a fixed statistical objective function. Unlike fixed objective functions (like those derived from OLS or GLM) that focus on explaining as much spending variation as possible, customized objective functions start from a theory on provider behavior and conceptualize RA as a tool to select weights that maximize its function (Ellis et al., 2018). Alternative estimation methods, such as constrained regression, can restrict weights to steer funds to undercompensated groups and accommodate the realization of an economic objective function (van Kleef et al., 2017, 2020)

### The Interconnection Between Design Choices for Risk Adjusters and Payment Weights

The presented framework covers several design questions on two main themes: (a) the choice of risk adjusters and (b) the choice of payment weights. It is important to emphasize that in practice, design choices both *within* and *between* these two themes are highly interrelated. Regarding design choices *within* the first theme, the choice of information that risk adjusters are based on will affect their specification and operationalization in such a way that trade-offs between selection and price/volume control are minimized. For example, the inclusion of a medication-based risk adjuster will likely require the consideration of conditions (i.e., thresholds) and restrictions for classification in order to minimize the risk of introducing incentives for new volume-related inefficiencies or perpetuating exiting ones, while maximizing fit and thus, minimizing selection-related incentives. Regarding design choices *within* the second theme, the choice of patient population and spending data affects the need for data modifications depending on the extent to which the observed/chosen population and data are representative for the population of interest to the payment model and their normative spending level in year *t*.

Design choices with respect to “how payment weights are derived” depend both on the choice of risk adjusters as well as on the choice of the (modified) estimation sample. In the ideal scenario, with access to a complete and unbiased set of C and R variables *and* an estimation sample that matches the population of interest and their normative spending level in year *t*, using standard/fixed optimization criteria (generally estimated through OLS) will determine accurate/unbiased payment weights without the need of additional corrections prior to estimation. Risks associated with increased technical complexity can then be avoided. In cases where the ideal scenario is violated, customized optimization criteria (and methods that accommodate these such as constrained regression or machine learning) might result in a better fit with the true objective function. In addition, addressing potential omitted-variable bias should be considered before estimation.

## Discussion

This article has developed an integral conceptual framework for the design of RA models in the context of prospective provider payment models in health care. As discussed below, we argue that there is no “one size fits all” approach in the design of RA models. This not only suggests that adequate RA design may differ across settings, but also that developments over time *within* a certain setting need to be incorporated, which can include technological advancements or changes in APM goals. In addition, successful application of this framework in practice requires not only a thorough understanding of the framework itself, but also consideration of various normative and practical aspects.

### Optimal RA Design Requires Separating C and R Variables

Deciding which variables are considered C and which R in RA models is a crucial normative decision that determines the source and degree of the risk to be adjusted for and therefore impacts the design choices of a RA model. However, although conceptually the distinction between C and R variables is clear, in practice the labeling as C or R of complex variables like preferences of uninformed patient groups that affect consumption patterns or discretionary quality-related variables that influence practice patterns is not straightforward and can vary depending on the context. In addition, C and R variables are not always directly observed in practice. Instead, we need to rely on imperfect “indicators” that might correlate with both C and R components. For example, diagnosis information is often used to estimate the C factor “health needs.” However, this information may also capture R signals such as a providers’ reporting quality, making it challenging to isolate the C component in observed spending levels and determine normative spending levels. Relatedly, we only observe *some* C and R variables, so it is likely that there exist some unobserved C variables that are associated with both the observed C and R variables but have not been taken into consideration. In this case, exclusion of the observed R variables may result in omitted-variable bias. Altogether, the accuracy of the compensation for C variables depends on the quality and the availability of the indicators in a particular context. Further research is needed to tackle the absence of a complete set of C and R variables and the potential biases that arise as a result.

### The Relative Weighting of Incentives Can Differ Across Contexts

The extent to which the potential selection and cost-control incentives are a concern can vary by context. To establish the extent to which perverse incentives are problematic for the design of a RA model, it is important to obtain a notion on the possible actions in terms of selection and cost control, their probability, and their potential effects in a given context. For instance, the use of diagnosis-based adjusters confronts the designers of the payment model with a trade-off between selection and cost control. In a context where options for selection are limited, the benefits of using such adjusters (i.e., less incentives for selection) may not outweigh the costs (i.e., less incentives for volume control). Conversely, in a context with many selection possibilities, the opposite may apply. In such a context, the benefits from including such adjuster (i.e., less incentives for selection) might *also* contribute to efficiency through the reduction of quality skimping (a potential negative consequence of risk selection). On the same note, less incentives for volume control, despite their negative effect on cost control, can also contribute to reducing incentives for quality skimping: by shifting the focus away from volume control, providers can use as much care as necessary for better quality.

Although it is possible to define potential actions related to selection and cost control, there is limited information on their probability and their effects in practice. One reason why this is poorly understood is that current evaluation metrics for selection and cost-control incentives have limitations. Specifically, current metrics like R-squared or Power (Geruso & McGuire, 2016) provide only general indication of selection and cost-control incentives and are based on statistical principles that poorly link to the economic objectives of provider payment systems. To truly understand these incentives, it is important to evaluate the seriousness of each incentive across patient groups, conditional to the possible provider actions and their underlying effects in a given context. Moreover, it is important to consider welfare/economic principles that extend beyond predicting the observed (and likely flawed) status quo. Overall, further exploration of such tailored welfare metrics is necessary to assess selection and cost-control incentives from RA.

### Feasibility Can Limit Optimal RA Design

The conceptual framework presented in this article is meant to serve as a basis for the design of a RA model in the context of APMs for health care providers. In practice, feasibility considerations such as data availability and stakeholder acceptability must naturally also be taken into account in the design of the model. Limited data availability, such as a small sample size and a lack of individual-level data, can compromise the stability and validity of the model. In addition, designers face the challenge of navigating the complexities of multiple design choices and options and ensuring their acceptability among the various stakeholders involved, especially among health care providers. Reaching a consensus among stakeholders on questions such as “what choices should be prioritized” can be challenging. In addition, regarding stakeholder acceptability, it is key that providers in particular understand the RA model and feel that the payment is fair and reflective of the unique characteristics of their patient population, as a lack of provider acceptability can prevent (active) participation and undermine the goals of the APM. All in all, the theoretically optimal design may not match with what is feasible in practical terms.

### Broader Considerations for RA Design in Health care Financing

In this article, we focused on the design of RA in the context of provider payment reform. However, provider payment reform is just one example of financing reforms aimed at improving efficiency. Other examples of such reforms are so-called “consumer engagement initiatives,” which include tiered cost-sharing and reference pricing (Dowd et al., 2021; Robinson et al., 2020). Although these reforms may also require RA and thus could benefit from the framework proposed in this article, further research is necessary to determine the extent of its applicability in these contexts.

In addition, although this article regards other relevant aspects or decisions related to health care financing (such as insurance coverage of care included in the payment scheme, or the inclusion of a pay-for-performance component to a prospective payment system) as given to maintain focus, it is important to acknowledge that in practice these aspects are also relevant for appropriate RA design. More research is needed into the role of all of these aspects and in the consequences thereof for RA design.

## Conclusion

The design of RA models for prospective provider payment systems is a complex exercise that requires explicit consideration of many difficult questions, choices, and trade-offs. This article has presented a conceptual framework summarizing these questions, choices and trade-offs, and provides several key insights. First, the design of RA models is not a one-size-fits-all approach and requires explicit normative decisions about which variables should be compensated for (C variables) and which should be providers’ responsibility (R variables). Second, the design process should be guided by three key criteria: (a) adequate compensation for spending variation due to C variables; (b) no compensation for spending variation due to R variables; and (c) feasibility. Third, the various design questions and options can be categorized along two overarching dimensions: (a) the choice of risk adjusters and (b) the choice of payment weights attached to these adjusters. It is important to explicitly make these choices in the light of the three criteria, considering the context of interest and the trade-offs between selection, cost control, and feasibility. Further research is necessary to support normative decisions regarding C and R variables, as well as develop comprehensive evaluation metrics for the assessment of incentives effects. Moreover, additional studies are needed to explore how other aspects of healthcare financing might impact RA design and to assess the overall applicability of our framework to different financing reforms.
